# Association of body composition and cardiovascular fitness with hypertension in a middle-aged adults: a cross-sectional study

**DOI:** 10.3389/fcvm.2025.1582936

**Published:** 2025-08-14

**Authors:** Junga Lee, Jisuk Chae, Mihee Kim, Soo Young Jung, Seung Don Yoo, Si-Hyuck Kang, Kyesan Lee, Jung-Hyun Kim

**Affiliations:** ^1^ Graduate School of Sport Science, Kyung Hee University, Yongin-si, Republic of Korea; ^2^ Department of Rehabilitation Medicine, Kyung Hee University College of Medicine, Kyung Hee University Hospital at Gangdong, Seoul, Republic of Korea; ^3^ Department of Internal Medicine, Seoul National University Bundang Hospital, Seongnam-Si, Republic of Korea; ^4^ Department of Electric, School of Electronics and Information, Kyung Hee University, Yongin-si, Republic of Korea; ^5^ Department of Sports Medicine, Kyung Hee University, Yongin-si, Republic of Korea

**Keywords:** hypertension, abdominal obesity, body composition, cardiovascular fitness, middle-aged adults, weight loss, step test

## Abstract

**Objectives:**

This study aims to investigate the association between cardiovascular fitness, body composition (particularly abdominal obesity), and hypertension in middle-aged adults, considering the influence of factors such as age, gender, smoking status and alcohol status.

**Methods:**

A cross-sectional study was conducted among 60 healthy adults (mean aged 54.23 ± 7.34) recruited. Participants underwent assessments of anthropometry, body composition, blood pressure, and physical fitness. The primary outcome was the prevalence of hypertension. Secondary outcomes included body composition measures (body fat mass, body lean mass and waist circumference), and physical fitness assessments [grip strength, sit-ups, sit-and-reach test, and Young Men's Christian Association step (YMCA) test]. Participants with hypertension had significantly higher body fat mass and waist circumference compared to those without hypertension.

**Results:**

The risk of hypertension was significantly increased by 16.8% with a 1 kg increase in body fat and by 14.0% with a 1 cm increase in waist circumference. The risk of hypertension was significantly increased in the group with hypertension accompanied by abdominal obesity as cardiovascular fitness, measured by the YMCA step test, decreased (Adjusted OR: 0.339, 95% CI: 0.170–0.679).

**Conclusion:**

These findings highlight the significant association between abdominal obesity, reduced cardiovascular fitness, and hypertension in middle-aged Korean adults. Abdominal obesity and low cardiovascular fitness were identified as independent risk factors for hypertension in this population. These results suggest that weight management to reduce abdominal obesity and regular physical activity to promote cardiovascular fitness are key to the prevention and management of hypertension.

## 1 Introduction

Hypertension is a significant global health problem that can lead to serious health complications, such as heart disease, stroke, and kidney disease (1, 2). Global hypertension cases more than doubled from 650 million in 1990 to 1.27 billion in 2019 World Health Organization (3). Middle-aged individuals are at higher risk due to lifestyle and hormonal changes, and poorly managed mid-life hypertension significantly increases cardiovascular disease risk and mortality in old age (4). Another 2024 study showed a 161.97% increase in hypertension-related chronic kidney disease prevalence since the 1990s, with over 1.57 million cases in 2019 alone (5). Hypertension is linked to brain blood vessel damage, increasing stroke and Alzheimer's risk (6–8). A significant positive association between hypertension and stroke was revealed by a decade-long study of U.S. adults (9), with hypertensive individuals found to be 3.9 times more prone to experiencing hemorrhagic stroke (10). Moreover, untreated hypertension is an independent risk factor for Alzheimer's compared to controlled hypertension or healthy controls (11). In fact, hypertension is a significant cause of death worldwide (12), and it imposes a huge socio-economic burden (13).

In spite of this seriousness, the importance of lifestyle modification in blood pressure management has been consistently stressed. A positive effect on blood pressure control has been demonstrated by numerous studies to be associated with regular physical activity. Aerobic exercise has been shown to be the most effective way to improve cardiovascular fitness (14, 15), and has a positive impact on hypertension management. Regular aerobic exercise has been shown to increase cardiac output as the heart's function is enhanced, the heart's workload is decreased, and blood pressure is reduced (16, 17).

Weight loss and reduction in body fat are indispensable components of hypertension management (18). Waist circumference and abdominal obesity have been strongly associated with the development of hypertension (19–23). High waist circumference has been shown to be a strong predictor of visceral adiposity, rather than a simple measure of overall body weight (24). Excessive nutrient intake has been shown to lead to enlarged visceral fat, which over time has been shown to damage tissues through endocrine stress, inducing inflammatory responses (25, 26), and disrupting glucose and lipid metabolism, thereby contributing to the development of hypertension (26, 27).

The specific mechanisms underlying these associations include the role of visceral fat in promoting insulin resistance and chronic inflammation, both of which are known to increase blood pressure (28, 29). Additionally, increased body fat is associated with sympathetic nervous system activation, contributing to elevated heart rate and vasoconstriction, further exacerbating hypertension (30).

While increased cardiovascular fitness, weight loss, and a reduction in body fat are essential components of hypertension management, current research lacks comprehensive studies that analyze the relationship between cardiovascular fitness, body fat, and hypertension. Additionally, several studies have investigated the associations between various physical fitness components, such as hand grip strength, sit-ups, step tests, sit and reach tests, and hypertension, independently (31, 32). However, specific analyses focusing on individual aspects of physical fitness are limited. One study examined several elements of physical fitness, including cardiovascular fitness, grip strength, sit-ups, sit and reach tests, vertical jumps, side steps, and one-leg standing with eyes open, in relation to hypertension in women (31, 32). The findings indicated that cardiovascular fitness, sit and reach scores, side steps, and one-leg standing were associated with hypertension. Conversely, a study conducted on the same cohort of men found a significant association only between sit and reach tests and hypertension. Another study compared the associations of cardiorespiratory fitness and muscle fitness with hypertension, finding that both were linked to hypertension (33). Regular aerobic exercise improves cardiovascular fitness by enhancing endothelial function and reducing arterial stiffness, which in turn lowers peripheral vascular resistance and blood pressure (34). Improved fitness also modulates the autonomic nervous system, reducing sympathetic activity and increasing parasympathetic tone, resulting in better blood pressure control (35).

Overall, the associations between each component of physical fitness and hypertension remain conflicting. Therefore, an investigation of the association between body composition, physical fitness including hand grip, sit-ups, step test, sit and reach test, and hypertension in middle-aged adults is warranted. This study investigates the relationship between abdominal obesity and cardiovascular fitness in relation to the occurrence of hypertension, providing insights into the mechanistic pathways that link these factors in middle-aged adults.

## 2 Methods

### 2.1 Study participants

The population of this study consisted of sixty middle-aged adults. The sample size calculation was based on a two-tailed test with a significance level of 0.05, a statistical power of 0.80, and an anticipated odd ratio (OR) of 5.1. Data were collected through anthropometric measurements and body composition analysis, physical fitness assessments, and resting metabolic rate measurements. Participants were recruited from May to July 2024 for this study. Inclusion criteria were as follows: (1) middle-aged Korean adults, (2) no clinical health problems that prevent the participant from engaging in exercise, and (3) no history of systematic exercise in the past six months. Exclusion criteria included: (1) pregnancy, (2) pre-existing musculoskeletal, cardiovascular, or immunological conditions that could impair exercise participation. Participants were recruited via poster advertisements displayed in the laboratory and distributed in online communities.

The Institutional Review Board (KHGIRB-23-468) approved this study, which was registered with the Clinical Research Information Service (KCT0010086), and conducted following established guidelines.

### 2.2 Experimental methods and procedures

#### 2.2.1 Pre-experiment preparation

**Figure 1:**
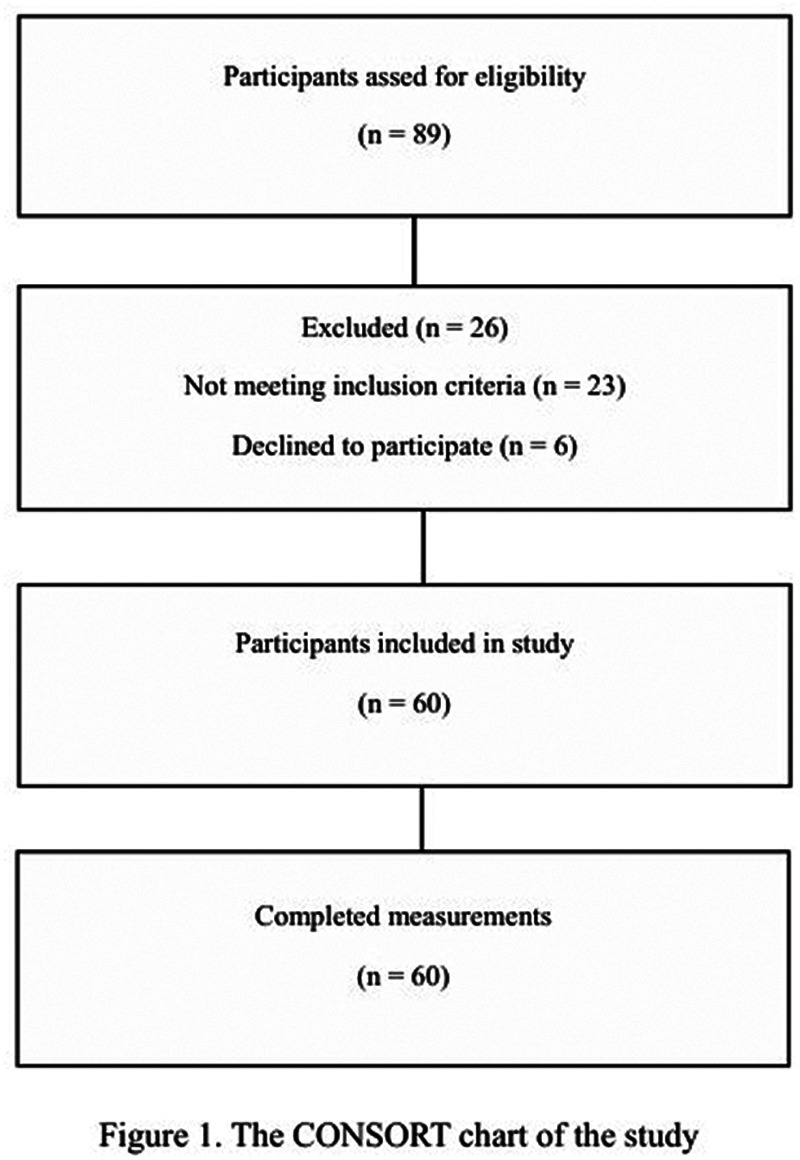
The CONSORT chart of the study.

Participants visited the laboratory once. One day prior to their visit, participants were instructed via phone to refrain from consuming food, alcohol, and caffeine for at least 8 h before the visit, and to avoid strenuous exercise for 24 h preceding the visit. After arriving, participants were informed about the study, provided consent, and were checked to ensure they met the study's requirements. Anthropometric measurements, body composition, physical fitness assessment and resting metabolic rate (RMR) were measured for all participants before the exercise. All measurements were performed using the same method by experienced researchers (Figure 1).

#### 2.2.2 Anthropometric measurements and body composition

Participants were instructed to wear comfortable clothing and athletic shoes for the physical fitness assessment. Anthropometric measurements, including height and weight, were obtained using a stadiometer (GL-150Tech, G-Tech International, Korea). Body mass index (BMI) was calculated as weight (kg) divided by height squared (m^2^). Waist and hip circumferences were measured using a tape measure, and the waist-to-hip ratio was calculated.

Blood pressure and heart rate were measured using an automated blood pressure monitor (JPN710T, OMRON, China). Participants were seated and allowed to rest for at least 10 min prior to measurement. The blood pressure cuff was positioned 2 cm above the antecubital crease of the left arm, at heart level. Systolic and diastolic blood pressures were recorded. Blood pressure was measured twice, and the average of these two readings was used.

Body composition, including body fat percentage and fat mass, were obtained using a bioelectrical impedance analysis device (Inbody 270, Biospace Inc, Korea).

#### 2.2.3 Physical fitness assessment

The National Fitness 100 is a national sports welfare program in Korea that offers exercise counseling and personalized prescriptions based on scientific evaluations and fitness assessments to enhance the health and fitness of the population. A nationwide assessment is conducted at 75 locations to evaluate the fitness levels of the general Korean population. In this context, the fitness measurement protocol of National Fitness 100 was employed in the study.

(1) Upper Body Strength

A dynamometer (TKK5401, TAKEI, Japan) was used to evaluate grip strength, which is an indicator of upper body strength. Participants assumed a standing posture with feet shoulder-width apart and grasped the handle with their dominant hand, ensuring a comfortable grip with the second joint of the fingers. The device's indicator was positioned facing outward. With arms fully extended and the torso inclined at a 15° angle, participants exerted maximum force on the handle for 3 s. This procedure was repeated twice for each hand, and the highest value was recorded to the nearest 0.1 kg. Relative grip strength was calculated using the following formula:

$$\left[Relative\;grip\;strength=\frac{grip\;strength(kg)}{Body\;weight(kg)}\times 100(\text{\%})\right]$$

(2) Muscular Endurance

Sit-ups were measured as an indicator of muscular endurance. Participants lay on a mat with their head and back flat, knees bent, feet flat on the floor, and feet hip-width apart. They placed their hands on their thighs with arms extended and performed sit-ups, touching the fingertips of the assessor who was holding their knees. The maximum number of repetitions in 1 min was recorded.

(3) Flexibility

The sit-and-reach test was used to assess flexibility. Participants sat on the floor with their legs extended and feet against a sit-and-reach box. They placed one hand on top of the other and reached forward as far as possible, holding the position for 2 s. This was repeated twice, and the highest value was recorded. Participants were instructed to avoid using momentum, bending their knees, or lifting their feet from the box.

(4) Cardiovascular Fitness

The YMCA step test was used to assess cardiovascular fitness. The YMCA step test was selected because of its practicality, ease of administration, and minimal equipment requirements, making it suitable for large-scale assessments and diverse settings (36). It effectively estimates cardiovascular endurance and provides a reliable measure of aerobic (37). Participants stepped up and down on a 30.5 cm step box at a rate of 96 beats per minute for 3 min. After 3 min, trained sports medicine researchers measured participants' heart rate in a seated position. Heart rate was recorded from the participants’ radial artery for 1 min. Maximal oxygen consumption was estimated using the following formula: This equation predicts a male and female's maximal oxygen consumption based on their age, height, weight, and heart rate recovery after one minute of exercise.

$$\begin{array}{rl} Male\;VO_{2}max & =70.597-0.246(age)+0.077(height)-0.222(body\;weight)\\ & \;-0.147(1\_\mathrm{minute}\;\mathrm{recovery}\;\mathrm{heart}\;\mathrm{rate}) \end{array}$$

$$\begin{array}{rl} Female\;VO_{2}max & =54.337-0.185(age)+0.097(height)-0.246(\\ & body\;weight)-0.122(1\_\mathrm{minute}\;\mathrm{recovery}\;\mathrm{heart}\;\mathrm{rate}) \end{array}$$

#### 2.2.4 Resting metabolic rate measurement

Before measuring resting metabolic rate (RMR), participants were asked to fast for 8 h and to avoid strenuous physical activity on the day of the measurement. Participants rested seated for 20 min before testing at the laboratory. A mask was fitted, and oxygen consumption and carbon dioxide production were measured for 15 min using a wireless gas analyzer (K5, Cosmed, Italy).

### 2.3 Data analysis

Data were analyzed using SPSS version 25.0. A statistical significance level of *p* < 0.05 was adopted throughout the study. The general characteristics of the participants were analyzed using descriptive statistics and frequency analysis.

Independent *t*-tests and chi-square or Fisher's exact tests were used to compare clinical characteristics between middle-aged Korean adults with and without abdominal obesity, as appropriate.

The association between hypertension diagnosis and body composition or abdominal obesity was analyzed using binary logistic regression analysis. This statistical method is employed when the dependent variable is presence of hypertension and the aim is to assess the influence of independent variables (body fat mass & skeletal muscle mass or waist circumferences) on the dependent variable.

The relationships between blood pressure, abdominal obesity, and physical fitness assessments (grip strength, sit-ups, sit-and-reach test, and YMCA step test) were analyzed using multinomial logistic regression. The dependent variables were blood pressure (normal blood pressure, prehypertension, hypertension) and abdominal obesity (normal waist circumference, abdominal obesity). The independent variables included measures of physical function and body composition.

By comparing the β coefficients and *p*-values across different groups, we set the significance level at 0.05.

## 3 Results

The general characteristics and physical fitness levels of the 60 middle-aged Korean adults who participated in this study are presented in Table 1.

**Table 1:** Clinical characteristics among the 40-69-year-old Korean adults.

Variables	Overall *n* = 60
Age (years)	54.23 ± 7.34
Male/Female, *n* (%)	30 (50)/30 (50)
Height (m)	1.66 ± 0.08
Weight (kg)	66.44 ± 14.27
BMI (kg/m^2^)	23.86 ± 3.99
Body Lean Mass (kg)	26.79 ± 6.22
Body Fat Mass (kg)	18.10 ± 7.48
Body Fat Percentage (%)	26.82 ± 7.63
Waist Circumference (cm)	88.75 ± 10.84
WHR	0.90 ± 0.06
Heart Rate (bpm)	70.70 ± 9.23
SBP (mmHg)	126.55 ± 16.45
DBP (mmHg)	81.12 ± 10.69
Self-reported smoking status	
Never, *n* (%)	54 (90)
Current, *n* (%)	6 (10)
Self-reported alcohol status	
Never, *n* (%)	31 (51.7)
Current, *n* (%)	29 (48.3)
Medication status	
Never	40 (66.7)
Thyroid	2 (3.3)
Hyperlipidemia	3 (5.0)
Hypertension	10 (16.7)
Cholesterol	5 (8.3)
Physical fitness componentS	
Hand grip (kg)	32.97 ± 9.92
Sit-ups (reps)	22.70 ± 11.24
Step test (ml/kg/min)	23.80 ± 5.50
Sit and reach test (cm)	10.69 ± 8.56
RMR (Kcal)	1,980.3 ± 394.38

Mean ± SD. *p*-values of the continuous variables were compared using independent samples t-tests. BMI, body mass index; DBP, diastolic blood pressure; RMR, resting metabolic rate; SBP, systolic blood pressure; WHR, waist to hip ratio.

The associations between hypertension diagnosis and body composition were examined in a binary logistic regression analysis, the results of which are presented in Table 2. A significant positive association was found between body fat mass and the risk of hypertension [Crude Odds Ratio (OR): 1.141, 95% CI: 1.029–1.266, Adjusted OR: 1.168, 95% CI: 1.013–1.347, *p* < 0.05], suggesting that individuals with greater body fat mass were at a higher risk of developing hypertension. However, no statistically significant association was found between skeletal muscle mass and hypertension diagnosis.

**Table 2:** Association between hypertension diagnosis and body composition.

Group	Variables	Crude ORs (95% CI)	Adjusted ORs (95% CI)
Normal (BP < 140/90 mmHg)	Body Fat mass (kg)	1 (reference)	1 (reference)
	Skeletal muscle mass (kg)		
Hypertension (140/90 mmHg ≤ BP)	Body Fat mass (kg)	1.141 (1.029–1.266)*	1.168 (1.013–1.347)*
	VIFs	1.54	1.62
	Skeletal muscle mass (kg)	1.042 (0.933–1.165)	1.163 (0.869–1.556)
	VIFs	1.32	1.43

Binary logistic regression analysis. Adjusted ORs are ORs adjusted for the confounding factor such as age, gender, smoking status and alcohol status. *p < 0.05, ^†^*p* < 0.01, and ^‡^*p* < 0.001. BP, blood pressure; VIFs, variance inflation factors.

A significant correlation between body fat and hypertension is demonstrated by Table 2, although it is indicated that skeletal muscle mass does not share the same correlation.

The results presented in Table 3 indicate that a significant positive association was found between waist circumference and the risk of hypertension. Binary logistic regression analysis demonstrated that a 1 cm increase in waist circumference was associated with a 14% increased odds of hypertension diagnosis. Even after adjusting for confounders, this association remained significant. The result suggests that abdominal obesity was found to be an independent risk factor for hypertension.

**Table 3:** Association between hypertension diagnosis and abdominal obesity.

Variables	Group	Crude ORs (95% CI)	Adjusted ORs (95% CI)
Waist circumference (cm)	Normal (BP < 140/90 mmHg)	1 (reference)	1 (reference)
	Hypertension (140/90 mmHg ≤ BP)	1.097 (1.028–1.170)^†^	1.140 (1.047–1.242)^†^
	VIFs	1.82	1.86

Binary logistic regression analysis. Adjusted ORs are ORs adjusted for the confounding factor such as age, gender, smoking status and alcohol status. *p < 0.05, ^†^*p* < 0.01, and ^‡^*p* < 0.001. BP, blood pressure; VIFs, variance inflation factors.

Table 3 is shown to demonstrate a significant correlation between waist circumference and hypertension.

The relationship between hypertension, abdominal obesity, and physical fitness components was examined using a multinomial logistic regression analysis, the results of which are presented in Table 4. These findings indicated that the relationship between physical fitness and hypertension was influenced by the presence of abdominal obesity. Among those with abdominal obesity, individuals in the prehypertension stage with lower step test scores were found to have a significantly increased risk of developing hypertension (Crude OR: 0.849, 95% CI: 0.655–1.102, Adjusted OR: 0.473, 95% CI: 0.263–0.849). Similarly, in the hypertension group, those with lower step test scores were found to have a significantly increased risk of hypertension (Crude OR: 0.670, 95% CI: 0.490–0.917, Adjusted OR: 0.339, 95% CI: 0.170–0.679). While no significant association was found between physical fitness and hypertension risk in those without abdominal obesity, this study results suggested that lower step test scores were significantly associated with a higher risk of hypertension in individuals with abdominal obesity. This implied that abdominal obesity strengthened the link between aerobic capacity and the risk of developing hypertension.

**Table 4:** Association between hypertension, abdominal obesity, and physical fitness components (grip strength, sit-ups, step test, and sit-and-reach test).

Group	Variables	Crude ORs (95% CI)	Adjusted ORs (95% CI)
Normal BP & normal WC	Hand grip (kg)	1	1
	Sit-ups (reps)		
	Step test (ml/kg/min)		
	Sit and reach test (cm)		
Normal BP & abdominal obesity	Hand grip (kg)	1.097 (0.959–1.256)	1.037 (0.788–1.365)
	VIFs	1.23	1.32
	Sit-ups (reps)	0.990 (0.889–1.102)	0.985 (0.882–1.099)
	VIFs	1.78	1.82
	Step test (ml/kg/min)	0.777 (0.594–1.017)	0.671 (0.416–1.081)
	VIFs	1.69	1.74
	Sit and reach test (cm)	0.968 (0.846–1.108)	0.957 (0.804–1.138)
	VIFs	1.72	1.78
Prehypertension & normal WC	Hand grip (kg)	1.030 (0.917–1.156)	1.084 (0.839–1.402)
	VIFs	1.34	1.38
	Sit-ups (reps)	0.965 (0.875–1.065)	0.939 (0.838–1.052)
	VIFs	1.42	1.51
	Step test (ml/kg/min)	1.096 (0.887–1.352)	1.192 (0.760–1.869)
	VIFs	1.53	1.60
	Sit and reach test (cm)	1.017 (0.901–1.148)	0.985 (0.846–1.146)
	VIFs	1.48	1.54
Prehypertension & abdominal obesity	Hand grip (kg)	1.137 (0.994–1.299)	0.905 (0.697–1.176)
	VIFs	1.72	0.81
	Sit-ups (reps)	0.958 (0.861–1.066)	0.992 (0.875–1.124)
	VIFs	1.83	1.91
	Step test (ml/kg/min)	0.849 (0.655–1.102)	0.473 (0.263–0.849)*
	VIFs	1.64	1.72
	Sit and reach test (cm)	0.957 (0.836–1.096)	0.974 (0.810–1.171)
	VIFs	1.88	1.93
Hypertension & normal WC	Hand grip (kg)	1.113 (0.903–1.373)	0.998 (0.659–1.511)
	VIFs	1.78	1.83
	Sit-ups (reps)	0.921 (0.783–1.082)	0.872 (0.672–1.132)
	VIFs	1.63	1.71
	Step test (ml/kg/min)	0.852 (0.570–1.274)	0.625 (0.273–1.432)
	VIFs	1.53	1.61
	Sit and reach test (cm)	1.067 (0.859–1.327)	1.171 (0.830–1.652)
	VIFs	1.81	1.89
Hypertension & abdominal obesity	Hand grip (kg)	1.224 (1.051–1.426)^†^	0.911 (0.673–1.234)
	VIFs	1.75	1.82
	Sit-ups (reps)	0.910 (0.802–1.033)	0.935 (0.799–1.095)
	VIFs	1.67	1.76
	Step test (ml/kg/min)	0.670 (0.490–0.917)*	0.339 (0.170–0.679)^†^
	VIFs	1.54	1.62
	Sit and reach test (cm)	0.939 (0.804–1.097)	1.002(0.812–1.237)
	VIFs	1.76	1.84

Multinomial logistic regression analysis. Adjusted ORs are ORs adjusted for the confounding factor such as age, gender, smoking status and alcohol status. *p < 0.05, ^†^*p* < 0.01, and ^‡^*p* < 0.001. BP, blood pressure; WC, waist circumference.

Normal BP: SBP < 120 mmHg and DBP < 80 mmHg.

Prehypertension: SBP 130–139 mmHg or DBP 80–89 mmHg.

Hypertension: 140 mmHg < SBP or 90 mmHg < DBP.

Table 4 shows that the step test results are significantly decreased in the hypertension and abdominal obesity groups.

The variance inflation factors (VIFs) for all independent variables, as presented in Tables 2–4, were found to be less than 2 in absolute value.

## 4 Discussion

The association between body fat mass, abdominal obesity, cardiovascular fitness, and hypertension in middle-aged adults was investigated in this study. It was found that hypertensive individuals had significantly higher body fat mass than those with normal blood pressure. The risk of hypertension was shown to be further increased by abdominal obesity, as well as by low cardiovascular fitness. This study found a significantly increased risk of hypertension associated with increasing body fat mass and waist circumference. After adjusting for confounders, each 1 kg increase in body fat was linked to a 16.8% higher risk of hypertension, and each 1 cm increase in waist circumference was linked to a 14.0% higher risk. Abdominal obesity was identified as a strong risk factor for hypertension. Supporting these findings, a 2018 study of diabetic patients in Shanghai also showed a significant positive correlation between abdominal obesity and the prevalence of cardiovascular disease and diabetic kidney disease, with increased visceral fat significantly increasing the odds of both conditions in both men and women (38). Inflammatory substances released by abdominal fat harm vascular endothelium, which led to increased atherosclerosis, impaired blood pressure regulation, and heightened heart strain, thus this could lead to heart failure (25). The heart is made to work harder to pump blood around the body by excess body fat, which can cause the heart muscle to thicken over time (39, 40). Cardiac hypertrophy, a thickened heart muscle is pumped harder, which raises blood pressure and puts more strain on blood vessels. This can contribute to the development of cardiovascular diseases, such as arteriosclerosis (39, 41). A recent study investigating the correlation between abdominal obesity and insulin resistance has revealed that visceral fat cells secrete inflammatory cytokines that contribute to insulin resistance (38, 42). Abdominal obesity could cause visceral fat accumulation and promote the secretion of inflammatory cytokines, which could impair vascular endothelial function and increase blood pressure (43, 44). A comprehensive review of research published before October 2022 indicated that adipokines, hormones produced by fat cells, play a central role in the inflammation associated with obesity. These hormones release substances that harm the inner lining of blood vessels, increasing the risk of conditions such as hardening of the arteries, high blood pressure, and heart disease (43). Also, adipose tissue contributed to elevated blood pressure by activating the renin-angiotensin-aldosterone system (45, 46). These findings are consistent with the proposed mechanism whereby visceral fat accumulation triggers insulin resistance, promotes the release of inflammatory cytokines, and activates the renin-angiotensin-aldosterone system, ultimately resulting in endothelial dysfunction and hypertension (42, 47).

This study was found to have individuals with low cardiovascular fitness at a higher risk of developing hypertension. After adjusting for age, gender, smoking status and alcohol status, the OR was 0.339 (95% CI, 0.170–0.679), indicating that the group with both hypertension and abdominal obesity had the lowest cardiovascular fitness. This suggested a direct link between decreased cardiovascular fitness and impaired blood pressure control. Several mechanisms contributed to the association between low cardiovascular fitness and an increased risk of hypertension. In a recent study of 138 overweight or obese adults, cardiovascular fitness was found to be associated with insulin sensitivity. Lower cardiovascular fitness was linked to reduced insulin sensitivity, which increased the risk of cardiovascular disease and type 2 diabetes (48). Low cardiovascular fitness can make it difficult for sufficient oxygen to be supplied during physical activity, which can cause tissue damage and chronic inflammation. This, in turn, can worsen vascular damage and lead to increased blood pressure (49). Excessive activation of the sympathetic nervous system due to a lack of physical activity can cause vasoconstriction and increase blood pressure (50). On the other hand, regular exercise has the effect of lowering blood pressure through the reduction of body fat, increased insulin sensitivity, and improved vascular endothelial function (51).

The finding that muscle strength and flexibility are not associated with hypertension can be attributed to several factors. Firstly, the limited sample size may have resulted in insufficient statistical power to detect a significant association. Additionally, behavioral factors such as physical activity levels, diet, and stress, which were not controlled for, might have influenced the results. Moreover, it is possible that muscle strength and flexibility are not directly related to hypertension, or that the relationship is mediated by other variables not measured in the study.

A previous meta-analysis study demonstrated that while resistance training might positively impact cardiovascular health in large population analyses, these effects are mainly associated with other health improvements such as reduced body fat (52). Another prior meta-analysis study found that poor trunk flexibility was associated with increased arterial stiffness compared to high flexibility (53). Furthermore, other studies comparing the effects of different types of physical activity on blood pressure have found that aerobic exercise is more effective in preventing hypertension than strength exercises (54). Therefore, it is important to consider the possibility that the effects of muscle strength and flexibility on hypertension are mediated by other intervention factors. Subsequent studies that control for various behavioral factors are needed, and a larger sample size would help to clarify these relationships more clearly.

A strong correlation has been found between abdominal obesity, hypertension, and cardiovascular health. The range of motion of the diaphragm is restricted by abdominal obesity, which led to a decrease in lung capacity. This affected cardiac output and blood oxygen saturation, and was considered a major factor that reduced cardiopulmonary function (55). Reduced cardiovascular fitness in individuals with both hypertension and abdominal obesity is indicated to be likely contributed to by multiple mechanisms, with these two conditions being considered primary drivers of this decreased function. The interplay between abdominal obesity, high blood pressure, and cardiovascular fitness is highlighted as a key determinant of health, requiring proper management. The need for health management strategies targeting obesity and low cardiovascular fitness is emphasized, with weight loss, healthy eating, and regular exercise being suggested as effective preventative and management tools for hypertension. Furthermore, the promotion of programs and campaigns focused on improving obesity and cardiovascular health through public health policies is advocated, ultimately fostering healthier lifestyles and enhancing overall public health.

This study revealed a relationship between the occurrence of hypertension and various factors, and several strengths were confirmed. First, a relationship between hypertension and various physical factors was explored in this study, and a strong correlation was revealed even after age, gender, smoking status, and alcohol status were controlled for. The potential role of improved cardiovascular fitness in hypertension management was also suggested. However, several limitations were identified. A small, non-representative sample of middle-aged Korean adults was used. Causal inference was prevented by the cross-sectional design. Furthermore, potential confounders such as diet, exercise habits, and genetic factors were not considered. It is recommended that larger, more diverse samples be employed in future research. Distributing poster advertisements not only in the laboratory but also in online communities can help expand the recruitment scope and attract a diverse range of participants. However, there are still limitations due to self-selection bias, lack of representativeness, and generalizability. Longitudinal designs and the incorporation of confounder and genetic analyses are also recommended for more robust conclusions about the relationship between hypertension, obesity, and physical activity. Medication information regarding participants' usage was not controlled when the participants were recruited, which may influence the results of this study. This should be considered when interpreting the results. Finally, the omission of important confounding variables such as diet, sleep patterns, habitual physical activity, and family history from the survey is a limitation. These factors could potentially impact the study results, and their exclusion suggests the possibility of residual confounding in the interpretation of the findings. Future research should include these variables to enable a more comprehensive analysis.

## 5 Conclusion

This study investigated the relationship between body fat, abdominal obesity, cardiovascular fitness, and hypertension in middle-aged adults. The findings indicate that increased body fat mass and abdominal obesity are significant risk factors for hypertension. Furthermore, abdominal obesity exacerbates the negative impact of hypertension on cardiovascular fitness, suggesting a synergistic relationship mediated by abdominal obesity. These results underscore the critical importance of lifestyle modifications, particularly focusing on weight loss and abdominal fat reduction, in managing hypertension.

In conclusion, the study demonstrates that achieving weight loss and enhancing cardiovascular fitness are essential strategies for the prevention and control of hypertension in middle-aged adults. Future research should focus on longitudinal studies to further elucidate the causal pathways and explore effective intervention strategies that incorporate dietary, behavioral, and physical activity modifications to mitigate the impact of these risk factors on hypertension.

## Data Availability

The raw data supporting the conclusions of this article will be made available by the authors, without undue reservation.
